# Albumin-Binding Domains in Therapeutic Protein Engineering: A Structural and Computational Perspective on Rational Design

**DOI:** 10.3390/synbio4010005

**Published:** 2026-02-12

**Authors:** Matthew J. Argyle, Dallin M. Chipman, Anna Claire Woolley, Bradley C. Bundy, Dennis Della Corte

**Affiliations:** 1 Department of Physics and Astronomy, Brigham Young University, Utah; 2 Department of Chemical and Biological Engineering, Brigham Young University, Utah

**Keywords:** albumin-binding domain, half-life extension, protein fusion, linker design, AlphaFold, structure prediction, therapeutic protein engineering, FcRn recycling

## Abstract

Therapeutic proteins face a critical pharmacokinetic challenge: rapid clearance from circulation limits their clinical efficacy. Albumin-binding domains (ABDs) offer an elegant solution by enabling therapeutic proteins to “hitchhike” on serum albumin’s favorable 19-day half-life through FcRn-mediated recycling. Clinical validation through approved therapeutics like ozoralizumab demonstrates the success of this approach, with preclinical studies showing fusion to an ABD extended half-life to 18 days. This review provides an analysis of ABD-fusion protein design, integrating structural biology, computational prediction, and rational engineering principles. We catalog the major classes of albumin-binding modalities, including bacterial three-helix bundle domains, engineered peptides, antibody-derived binders, and alternative scaffolds, comparing their binding properties, size contributions, cross-species reactivity, and production cost. Critical examination of linker architectures reveals that flexible glycine-serine linkers (particularly the widely successful (GGGGS)_3_ motif) provide optimal balance between domain independence and molecular economy, though linker choice profoundly influences not only spatial separation but also binding affinity, folding, stability, and pharmacokinetics. We evaluate the utility and limitations of the structure prediction tools for ABD-fusion design. We establish practical guidelines for integrating computational screening with experimental validation. This review provides protein engineers and synthetic biologists with a comprehensive framework for rational design of albumin-binding therapeutics, emphasizing the synergistic integration of structural insight, computational prediction, and systematic experimental validation to accelerate development of next-generation long-acting biotherapeutics.

## 1. Introduction

The development of therapeutic proteins and peptides has revolutionized modern medicine, offering unprecedented specificity for molecular targets implicated in human disease. However, the clinical translation of these biologics faces a fundamental pharmacokinetic challenge: protein and peptide-based therapeutics suffer from poor circulation half-life in vivo, often measured in minutes rather than the hours or days required for therapeutic efficacy [1]. This limitation stems from rapid renal filtration of molecules below approximately 60–70 kDa, proteolytic degradation, and clearance by the reticuloendothelial system [2]. The clinical consequences are substantial—frequent dosing regimens reduce patient compliance, increase healthcare costs, and limit the therapeutic window [3]. For example, native glucagon-like peptide-1 (GLP-1), a potent incretin hormone for diabetes treatment, exhibits a half-life of a rather short 2 minutes, necessitating continuous infusion for therapeutic effect [4].

**Figure 1.**
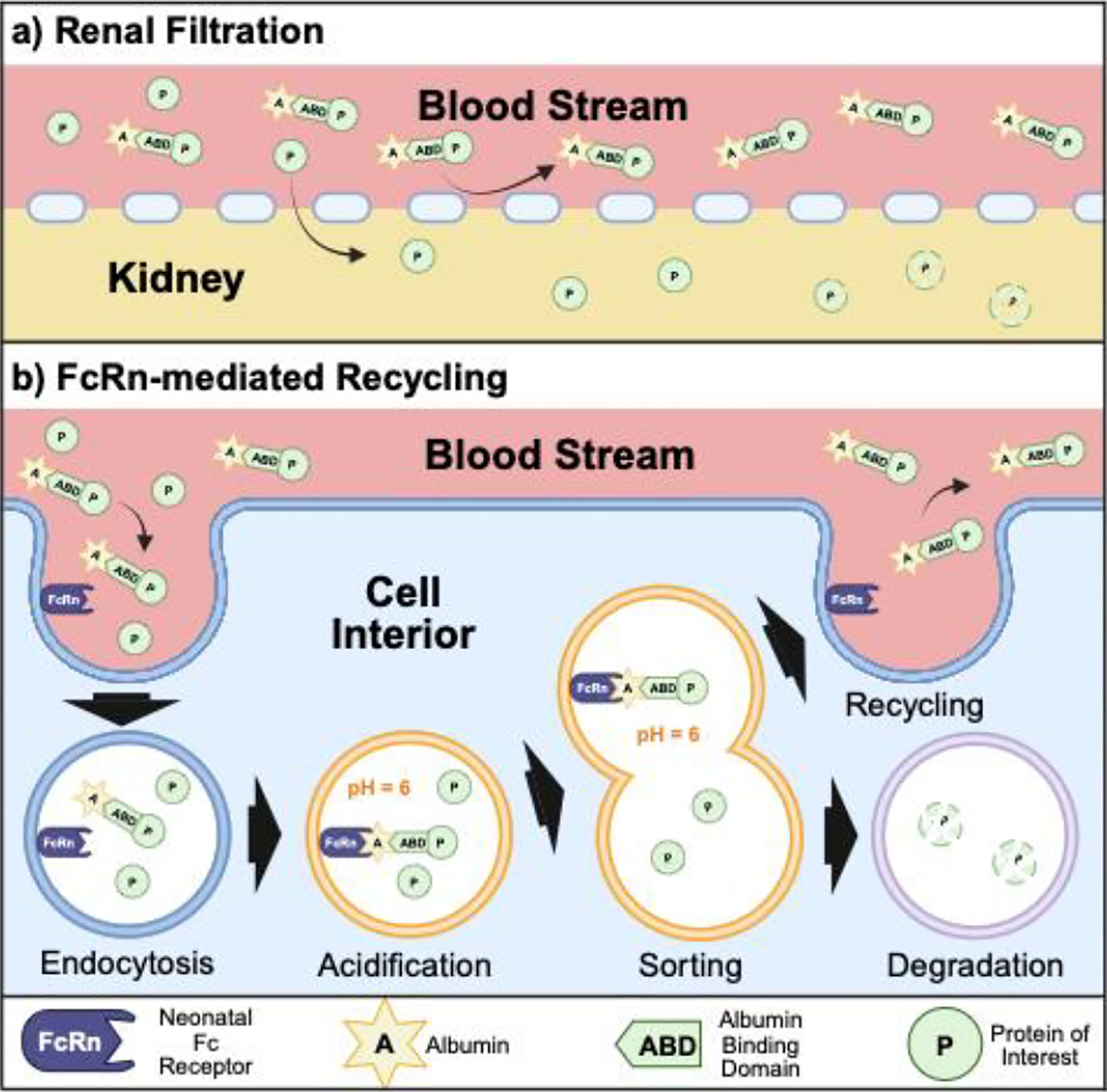
Mechanisms underlying albumin-mediated extension of protein half-life. a) Albumin exceeds the molecular size threshold for glomerular filtration, thereby conferring renal protection to conjugated therapeutic proteins. b) Albumin undergoes FcRn-mediated recycling via pH-dependent binding in acidified endosomes, rescuing both albumin and its conjugated cargo from lysosomal degradation. Created in BioRender. Bundy, B. (2026) https://BioRender.com/geufpx6

Evolution has provided an elegant solution to the half-life problem in the form of human serum albumin (HSA). At concentrations of 35–50 g/L, albumin represents the most abundant protein in human plasma [5]. Albumin’s half-life in the body is a lengthy 19 days. This is made possible because albumin can escape both renal filtration and lysosomal degradation (see Figure 1). Albumin is largely excluded from renal filtration due to the size and charge-selective properties of the glomerular filtration barrier, whose fenestrated endothelium, basement membrane, and podocyte slit diaphragms collectively restrict the passage of macromolecules exceeding approximately 3–4 nm in radius [2,6]. The glomerular capillary wall barrier also possesses a negative charge which repels albumin, further explaining why less than 0.001% of albumin gets filtered out of the body through the kidneys [7]. Any protein – even therapeutics – bound to albumin inherits these abilities and is similarly protected from renal filtration. Additionally, albumin can take advantage of the neonatal FC receptor (FcRn) recycling pathway, which allows albumin to bypass lysosomal degradation [8]. The albumin recycling mechanism mediated by FcRn represents a sophisticated biological transport system: following endocytosis, albumin binds FcRn in acidic endosomal compartments (pH approximately 6.0) and is returned to the cell surface, where it dissociates from FcRn at physiological pH (approximately 7.4) and re-enters circulation [9,10]. Fusing proteins to an ABD has demonstrated dramatic improvements in pharmacokinetic properties, with the 10 kDa protein Tencon25 increasing its half-life in mice from 40 minutes to 60 hours upon addition of an ABD [11].

The therapeutic exploitation of albumin’s properties has evolved through several conceptual phases. Early approaches involved direct chemical conjugation of drugs to HSA or formulation with albumin as a carrier, exemplified by nab-paclitaxel (Abraxane^®^) [12]. A more sophisticated strategy emerged with the discovery of albumin-binding domains (ABDs) in bacterial surface proteins. These compact domains, particularly those derived from streptococcal protein G and protein PAB from Finegoldia magna, exhibit high-affinity binding to albumin through defined structural interfaces [8]. Their small size (typically 5–7 kDa), high stability, and characterized binding mechanisms made them attractive candidates for genetic fusion to therapeutic proteins. In parallel, pharmaceutical chemistry approaches developed fatty acid conjugation strategies, wherein long-chain fatty acids serve as albumin-binding moieties. This approach, exemplified by drugs such as liraglutide and semaglutide, demonstrates the clinical viability of albumin-mediated half-life extension [13]. More recent innovations have introduced additional albumin-binding modalities, including perfluoroaryl-modified macrocyclic peptides and engineered nanobodies that bind albumin while retaining compact size [14,15]. Ozoralizumab, a drug consisting of nanobodies targeting TNF-α and human albumin, has an impressive half-life of 18 days. This favorable pharmacokinetic profile has helped ozoralizumab obtain approval for treatment of rheumatoid arthritis in Japan, demonstrating the validity of albumin binding for therapeutic approval [16].

While the importance of albumin binding for half-life extension is well-established, an equally critical yet often underappreciated component of successful ABD-therapeutic protein fusions is the molecular linker connecting these domains. The linker serves multiple functions beyond simple covalent attachment: it must provide sufficient spatial separation to allow independent folding of each domain, permit the conformational flexibility necessary for albumin engagement, and avoid creating new interaction surfaces that could compromise either the binding affinity of the ABD or the biological activity of the therapeutic protein [17–19]. Linker architectures described in the literature range from highly flexible glycine-serine repeats, which maximize conformational freedom, to rigid proline-rich or helical structures that maintain fixed spatial relationships between domains [20]. The choice of linker is not merely a technical detail but a critical design parameter that can determine the success or failure of a fusion protein therapeutic.

The advent of artificial intelligence in structural biology has revolutionized our ability to predict and optimize protein architectures. AlphaFold and its derivatives have achieved remarkable accuracy in protein structure prediction, enabling incredible advances in protein design and drug discovery [21,22]. The AlphaFold Database now provides access to over 214 million predicted protein structures; although not all predictions are accurate, the models include confidence scores that enable assessment of structural reliability [23]. For ABD-linker-therapeutic protein fusions, computational predictions can quickly provide an initial idea of an answer to several key questions: Does the linker provide adequate spatial separation? Are there unexpected inter-domain interactions? Is the ABD-albumin binding interface preserved in the fusion context? However, despite being useful as a starting point, computational predictions have important limitations that must be acknowledged. AlphaFold3 can struggle to predict some protein ligand interactions, correctly fold novel proteins or proteins that are underrepresented in the training data, and model intrinsically disordered regions [24]. Nevertheless, when used judiciously and in combination with necessary experimental validation, AI-driven structure prediction represents a powerful tool in the rational design toolkit.

In this review, we catalog the known classes of albumin-binding domains, comparing their structural characteristics, binding mechanisms, and practical advantages. We then surveyed the landscape of linker architectures employed in protein fusions, examining how they influence fusion protein behavior. We conclude by synthesizing general principles for rational design of ABD-linker-therapeutic protein fusions, providing synthetic biologists and protein engineers with a framework for approaching ABD fusion design through both experimental iteration and computation-guided rational design.

## 2. ALBUMIN-BINDING DOMAINS: A STRUCTURAL CLASSIFICATION

The landscape of albumin-binding domains encompasses diverse structural classes, each exploiting different molecular recognition strategies to achieve high-affinity binding to serum albumin. This section provides a catalog of common albumin-binding modalities. Although extensive research has been reported on albumin nanoparticles and inorganic nanoparticles coated with albumin, these have primarily been used for drug or gene delivery applications and have been excluded from this section focused on albumin additions to a therapeutic protein to increase retention [25–27].

### 2.1 Bacterial Three-Helix Bundle Domains

The most extensively characterized albumin-binding domains originate from bacterial surface proteins, where albumin binding plays a role in pathogenesis and immune evasion. Many protein engineering approaches have been based on the related domains of streptococcal protein G and protein PAB from Finegoldia magna. The most thoroughly studied domains of each of these proteins are referred to as GA and as G148-ABD, derived from streptococcal protein G and PAB, respectively. G148-ABD, consisting of only 46 amino acids, represents the minimal functional unit for albumin binding [8]. These domains adopt a compact three-helix bundle architecture, with helices arranged in an up-down-up topology around a hydrophobic core [8]. The binding interface for these domains has been mapped through crystallographic studies, revealing that specific residues in helix 2 and the surrounding loops make critical contacts with domain II of albumin [31]. The albumin binding site has no overlap with albumin’s FcRn-binding region in domains I and III, allowing ABD-albumin complexes to undergo FcRn-mediated recycling [8].

Through affinity maturation efforts, engineered variants have been developed with dissociation constants reaching as low as 10^−^^1^^3^ M for binding to human serum albumin, representing some of the highest affinities achieved for non-covalent protein-protein interactions [8]. Key engineered variants include ABD035, a variant with high cross-species affinity for both human and mouse serum albumin [35]; ABDCon, a consensus-designed variant with exceptional stability (melting temperature of 81.5°C) and picomolar affinity (61 pM) for human albumin [11]; and ABD23, a truncated variant incorporating only the core albumin-binding residues, which maintains comparable binding affinity despite reduced size and helicity [8,31,36].

Bacterial three-helix bundle ABDs offer several advantages for therapeutic applications: small size (typically 5–7 kDa), high thermostability, lack of disulfide bonds facilitating cytoplasmic expression, and well-characterized binding mechanisms (see Table 1). However, potential concerns include residual immunogenicity in human applications, necessitating deimmunization efforts such as those that produced the ABD094 variant, which shows no immunogenic potential in T-cell proliferation assays [8].

### 2.2 Engineered Peptide-Based Binders

Complementing the folded protein domains, short peptide sequences have been developed that bind albumin through mechanisms distinct from the three-helix bundles. Novel albumin-binding macrocyclic peptides have been selected from genetically encoded libraries using perfluoroaryl-cysteine chemistry, with lead sequences exhibiting micromolar affinity for human serum albumin [14]. These peptides typically range from 4–24 amino acids and rely on conformational constraint through cyclization or stapling to present binding determinants. As a consequence of their short sequence length and constrained architecture, such macrocyclic peptide binders generally contribute less than ~2 kDa of additional molecular weight [37].

Peptide-based binders offer minimal size addition, straightforward chemical synthesis, and compatibility with various conjugation chemistries. However, they generally exhibit lower binding affinities than folded domains (micromolar versus nanomolar or picomolar), may show reduced stability, and can present challenges in maintaining consistent binding in different fusion contexts [38].

While technically not protein domains, fatty acid modifications also deserve mention as albumin-binding moieties. Albumin’s natural affinity for fatty acids has led to the development of fatty acid-based ABDs [39]. Long-chain fatty acids (C14-C20) bind to multiple sites within albumin’s lipid-binding pockets, providing an alternative strategy for albumin association [40]. When conjugated to peptides, these fatty acids increase the overall molecular weight of the construct, so peptide–fatty acid conjugates typically range from ~3–5 kDa depending on the length of the lipid and any linker chemistry used [41]. This approach has proven particularly successful for small peptides like insulin or GLP-1, where fatty acid acylation enables once-weekly dosing regimens [5,13,42]. By fusing therapeutics to fatty acids native to the body there is the additional benefit of lower risk of an immune response [43]. They are, however, only attachable to the desired protein through chemical synthesis, which can increase expression difficulty [44,45].

### 2.3 Antibody-Derived Albumin Binders

The immunoglobulin scaffold has been exploited to generate albumin-binding moieties that combine high affinity with the favorable properties of antibody frameworks. Camelid-derived single-domain antibodies (VHH domains or nanobodies) that bind albumin have been developed through immunization and library selection approaches. These approximately 15 kDa domains maintain the robust immunoglobulin fold while providing a compact, single-chain format [46,47]. Engineered “knob domains” derived from bovine ultralong CDR H3 regions represent some of the smallest antibody-derived fragments capable of antigen binding, and these have been engineered to bind albumin and integrated into various therapeutic formats [15]. Remarkably, bispecificity has been engineered into single albumin-binding domains, yielding minimal 46 amino-acid proteins capable of simultaneously engaging albumin and a therapeutic target like TNF-α [48]. In addition, VHH-based albumin binders display high intrinsic thermal stability, retaining their folded structure and binding function even after exposure to elevated temperatures [49]. Together, these features exemplify an important minimization strategy for the design of robust albumin-binding therapeutics.

Antibody-derived binders benefit from well-understood immunoglobulin properties and generally low immunogenicity, particularly for humanized or human frameworks. However, they are larger than minimal ABD domains (15 kDa versus 5–7 kDa), require disulfide bonds that complicate some expression systems, and may be more expensive to produce than bacterial-derived domains.

### 2.4 Comparative Analysis and Selection Criteria

The choice among albumin-binding domain classes depends on multiple factors that must be carefully balanced for each therapeutic application. Regarding binding affinity, three-helix bundle bacterial ABDs achieve nanomolar to picomolar range, antibody-derived binders achieve nanomolar range, and peptide binders achieve micromolar to nanomolar range. Size considerations are equally important: minimal peptides contribute less than 2 kDa, bacterial ABDs contribute 5–7 kDa, and antibody domains contribute 12–15 kDa to the overall fusion protein mass.

Not all albumin binders show equivalent affinity across species, which is critical for preclinical development. Engineered bacterial-based variants like ABDCon have been optimized for binding to human, monkey, and mouse albumin, facilitating preclinical-to-clinical translation [11]. This cross-species reactivity is particularly valuable for pharmacokinetic studies in animal models. Engineered ABDs can also achieve remarkable thermostability, with melting temperatures exceeding 80°C, which is advantageous for formulation, storage, and production processes [11]. While protein therapeutics always carry some risk of immunogenicity, choices can minimize this risk through selection of humanized antibody frameworks or deimmunized variants of bacterial ABDs developed through substitution of immunogenic epitopes [8,53]. Production considerations also significantly impact manufacturing costs and scalability—bacterial ABDs lacking disulfide bonds can be produced in E. coli cytoplasm with high yields, while antibody-derived domains typically require periplasmic expression or eukaryotic systems for proper disulfide formation.

## 3. LINKER ARCHITECTURES IN PROTEIN FUSIONS

The molecular linker connecting an albumin-binding domain to a therapeutic protein represents far more than a simple tether between functional modules. Linkers profoundly influence fusion protein folding, stability, pharmacokinetics, binding properties, and therapeutic efficacy. This section reviews the classes of linkers employed in protein fusions, with emphasis on design principles relevant to ABD-therapeutic protein constructs.

### 3.1 Flexible Glycine-Serine Linkers

Flexible linkers represent the most employed class in fusion protein design, characterized by high glycine and serine content that provides conformational freedom while maintaining hydrophilicity [55]. The archetypal flexible linker follows the (GGGS)_n_ or (GGGGS)_n_ motif, where n determines the overall linker length [56–59]. These linkers provide the required physical separation between the domains that allows each domain to both fold correctly during expression and to independently bind to its respective targets [17] [17]. Glycine, lacking a side chain beyond hydrogen, imposes minimal steric constraints on backbone conformation and thus increases flexibility, while serine provides hydrophilicity and potential for hydrogen bonding with surrounding solvent [55]. The flexible glycine/serine linker consistently proves to present little to no risk of immunogenic response and is thus a popular choice for FDA-approved therapeutics [60].

Linker length emerges as a critical variable with functional consequences extending beyond simple spatial separation. Short linkers of 5–10 amino acids minimize the size addition but may constrain domain mobility, potentially limiting conformational sampling necessary for optimal binding. Medium linkers of 10–20 amino acids represent the most common length range, balancing flexibility with manageable size. Long linkers exceeding 20 amino acids maximize domain independence but increase molecular weight and may introduce aggregation propensity or provide sites for proteolytic cleavage [61–63]. In the development of GLP-1-ABD-XTEN fusion proteins, a (GGGGS)_3_ flexible linker was employed both between GLP-1 and ABD and between ABD and XTEN to allow proper domain folding and independent receptor binding [17]. This 15-residue motif appears repeatedly in successful designs across diverse therapeutic proteins, suggesting this length provides a favorable balance for many applications.

A few engineered proteins have used XTEN as a flexible linker between protein domains [64–66]. XTEN is a long, unstructured chain of amino acids typically used as a protein extension to increase hydrodynamic radius and provide shielding from immunogenic effects [67]. It has been reported to increase half-life when used as a linker as well [68].

In computational structure prediction, flexible linker regions typically show lower confidence scores and often exhibit disordered or extended conformations in predicted models. This behavior reflects their genuine structural plasticity rather than prediction failure—experimentally, flexible linkers exist as conformational ensembles rather than single defined structures [69]. This property is both advantageous, allowing domain orientation flexibility, and challenging, complicating structural characterization. The practical advantages of flexible linkers include their ability to minimize domain-domain interactions, their simple design and implementation, their general tolerance across diverse fusion contexts, and their low immunogenic potential. However, they may introduce unwanted flexibility leading to reduced effective concentration for bivalent constructs, they can serve as proteolytic cleavage sites, and their extended conformations may increase hydrodynamic radius beyond that expected from molecular weight alone.

### 3.2 Rigid and Semi-Rigid Linkers

In contrast to flexible linkers, rigid linkers maintain defined secondary structures, typically α-helices or extended polyproline II helices, that constrain the relative orientation of connected domains. Sequences with high helical propensity (rich in alanine, leucine, and glutamate) can form stable α-helices that function as rigid spacers, maintaining a defined spatial relationship between domains. This can be particularly beneficial when precise geometric arrangements are required for optimal function [20]. Proline-rich motifs such as (XP)_n_, where X is typically alanine or other small residues, adopt polyproline II helix conformations—extended, relatively rigid structures with approximately 3 Å per residue rise that provide linear separation between domains with minimal bending [70,71].

Rigid linkers find application when specific domain orientations are required for function, when minimizing entropic costs of binding is beneficial, or when preventing domain occlusion is critical. However, the reduced conformational freedom can be disadvantageous if the optimal relative domain orientation is unknown or if different orientations are required for different steps of the mechanism, such as albumin binding versus target engagement.

### 3.3 Cleavable Linkers

Cleavable linkers incorporate specific recognition sequences for proteases or chemical instability under physiological conditions, enabling controlled release of the therapeutic domain from the ABD-albumin complex [72–74]. These linkers can be designed for cleavage by extracellular proteases enriched in disease microenvironments (e.g., matrix metalloproteinases in tumors), intracellular proteases following endocytosis (e.g., cathepsins in lysosomes), or engineered proteases for controlled activation. For example, acid-sensitive hydrazone linkers have been employed in albumin-drug conjugates, designed for cleavage in acidic tumor microenvironments or within endosomes following cellular uptake [5].

Cleavable linkers enable sophisticated therapeutic strategies: prolonged circulation in ABD-bound form with release at disease sites, protection of labile therapeutic domains during circulation, and controlled activation of prodrug-like constructs. However, premature cleavage during circulation can compromise pharmacokinetics, and cleavage products must be carefully evaluated for safety and efficacy.

### 3.4 Impact of Linker Choice on Protein Function

The choice of linker architecture exerts multifaceted effects on fusion protein behavior, extending well beyond simple connectivity. Systematic studies comparing different linker entities in PSMA-targeting radioligands containing albumin binders revealed that linker structure directly impacts albumin-binding affinity, demonstrating that linkers are not passive spacers but active determinants of binding properties [75]. This effect may arise through linker participation in or near the binding interface, influence on ABD conformational dynamics, alteration of the effective local concentration for binding, or steric and electrostatic effects on domain approach. Linker choice influences whether domains fold independently or co-dependently during biosynthesis. Flexible linkers generally promote independent domain folding, while short or rigid linkers may require coordinated folding that could be less thermodynamically favorable. Beyond the direct effects of ABD-albumin binding, linkers influence pharmacokinetics through alteration of hydrodynamic radius (affecting renal filtration), susceptibility to proteolytic degradation in circulation, impact on tissue distribution and cellular uptake, and effects on clearance mechanisms independent of albumin binding. Linker hydrophobicity and charge distribution significantly impact aggregation propensity, a critical consideration for therapeutic development. While linkers are often considered non-immunogenic due to their simple, repetitive sequences, junction regions where linkers meet functional domains can create novel epitopes that should be evaluated during design optimization.

## 4. STRUCTURAL COMPARISON: EXPERIMENTAL AND COMPUTATIONAL APPROACHES

The integration of experimental structural biology with computational prediction methods represents a transformative opportunity in therapeutic protein engineering [76,77]. This section provides analysis of how experimentally determined structures of albumin-binding domains compare with computationally predicted models, with particular emphasis on evaluating the utility and limitations of AlphaFold for rational design of ABD-fusion protein therapeutics.

### 4.1 Experimental Structures of ABDs and Complexes

The structural characterization of albumin-binding domains has been extensively pursued through nuclear magnetic resonance (NMR) spectroscopy and X-ray crystallography. The first three-dimensional structure of an albumin-binding domain was determined by NMR for the GA module from protein PAB of Finegoldia magna, revealing a compact left-handed three-helix bundle architecture comprising approximately 45 amino acid residues (PDB: 1PRB) [78]. This pioneering structural study established the canonical fold for this class of binding domains and demonstrated remarkable thermal stability despite the small size.

Subsequent structural studies of the albumin-binding domain from streptococcal protein G confirmed the conservation of the three-helix bundle architecture across homologous sequences. NMR solution structures (PDB: 1GJS, 1GJT) revealed that despite only 59% sequence identity, both domains adopt nearly identical overall folds [50]. The most informative structural insights have come from crystal structures of ABD-albumin complexes. The crystal structure of the GA module from protein PAB in complex with human serum albumin, clarified to 2.7 Å resolution (PDB: 1TF0), revealed that the GA module binds to an epitope in domain II of albumin [31]. This structure definitively established that the ABD binding site does not overlap with the FcRn-binding region on albumin, explaining how ABD-albumin complexes can undergo efficient FcRn-mediated recycling. Additional crystal structures with a bound fatty acid and a drug molecule (PDB: 2VDB) demonstrate the conformational plasticity of albumin and the ability of GA to bind to multiple of these conformations [79]. This has important implications for computational prediction; a single static structure may not fully represent the binding-competent ensemble.

### 4.2 Computational Prediction of ABD-Albumin Complexes and Binding Interfaces

AlphaFold3 has been revolutionary for computational protein engineering. It achieves high accuracy for albumin-binding domain structure prediction, with backbone RMSDs typically below 1.5 Å for the core three-helix bundle architecture [80,81]. For high-confidence regions with per-residue confidence scores (pLDDT) above 90, the median RMSD approaches 0.6 Å, comparable to variability between experimental structures. However, limitations exist for flexible regions. AlphaFold3 has been found to predict loop regions to withing 2 Å accuracy only about 40% of the time, although other methods designed specifically for loop regions approximately double this success rate [82]. These loop regions are particularly relevant for ABD inter-helical loops critical for albumin binding.

For protein-protein complex prediction, AlphaFold3 shows enhanced performance over earlier versions. In a comparative study with 11 prediction softwares, AlphaFold showed the most accuracy when apo initial conformations were used, with a prediction success rate of over 70%. These predictions correctly predict the binding interface with sub-angstrom accuracy [83]. The interface prediction metric (ipTM score) provides confidence assessment—scores above 0.8 indicate highly confident interface geometry predictions, while scores below 0.6 suggest the predicted interface is likely incorrect. Importantly, binding interface residues on both albumin and ABD are generally predicted with moderate to high confidence in isolation, enabling reliable interface design even when complex prediction is uncertain. Side chain prediction shows approximately 7% of residues incompatible with experimental data, requiring validation for binding interfaces where precise orientation determines affinity. AlphaFold3 is also able to sample different confirmation states of some proteins which help make predictions more accurate [84].

### 4.3 Linker Prediction Challenges

Flexible linkers represent a fundamental challenge for structure prediction as they exist as conformational ensembles rather than single structures. AlphaFold3 predictions of glycine-serine linkers typically show low pLDDT scores (50–70) and extended conformations representing one possibility from a large ensemble rather than definitive structures. The predicted aligned error (PAE) metric reveals domain-domain relationships—high PAE values (>15–20 Å) between domains connected by flexible linkers correctly indicate uncertain relative orientations, even when individual domains show high confidence.

RFdiffusion offers a complementary approach by designing structured linkers with defined geometries when specific domain orientations are functionally required. Using diffusion models, RFdiffusion can generate linker sequences that optimize domain spacing, minimize steric clashes, and position therapeutic domains for optimal activity. This provides an alternative to flexible linkers when precise spatial control is needed while maintaining designability [85,86].

### 4.4 Confidence Metrics as Design Tools

AlphaFold3 confidence metrics guide design decisions when properly interpreted. The pLDDT score provides a first-pass filter: regions above 90 are accurate within 1–2 Å for backbone atoms and suitable for detailed interface analysis; regions between 70–90 indicate good backbone confidence but greater side chain uncertainty; regions below 70 indicate substantial uncertainty and should be treated as hypothetical models, though for flexible linkers these low scores correctly reflect genuine disorder.

PAE metrics complement pLDDT by revealing relative positioning confidence. Low PAE (<5 Å) indicates confident relative positioning, while high PAE (>15–20 Å) indicates uncertainty even when individual regions have high pLDDT. For fusion proteins, PAE plots typically show high confidence within domains but high uncertainty across linkers, validating flexible linker design principles.

Confidence scores cannot predict all functionally relevant aspects—they do not account for post-translational modifications, ligand-induced conformational changes, pH-dependent transitions, or expression and aggregation behavior. These limitations necessitate integration with experimental validation [87].

### 4.5 Integrating Computational Optimization and Experimental Approaches

The most effective strategy integrates computational prediction with experimental validation in iterative cycles [88]. A recommended workflow begins with computational screening: using AlphaFold3 to assess independent domain folding and predict ABD-albumin interface geometry (prioritizing ipTM scores >0.8), applying RFdiffusion to design optimized linker architectures for appropriate flexibility or structured geometries, and employing BayesDesign, a probabilistic inverse folding method, to optimize sequences for thermostability and conformational specificity while maintaining binding function. These screens can help identify candidates and potential design issues before experimental work [89]. Because albumin binding has been reported to vary with buffer conditions such as ionic strength and pH, pre-expression screens should also be conducted at the conditions of the intended application [90,91].

For designs passing computational filters, initial low-resource experimental assessment, such as pilot expression, solubility screening, and preliminary albumin-binding assays, should be conducted before committing to resource-intensive validation. Subsequent characterization will then focus on detailed biophysical analysis of stability and aggregation, albumin-binding affinity confirmation, and therapeutic domain activity assessment. When experimental results deviate from predictions, discrepancies provide valuable learning opportunities for refining computational models. As computational methods advance, improving flexibility prediction, complex modeling accuracy, and prediction of stability and expression properties, the synergy between computation and experiment will strengthen, accelerating albumin-binding therapeutic development.

## 5. Conclusions

Bacterial three-helix bundle ABDs derived from streptococcal protein G and F. magna protein PAB provide robust therapeutic scaffolds combining small size (5–7 kDa), exceptional thermal stability (Tm ~70–85°C), high binding affinity (femtomolar to nanomolar range), low immunogenic potential, and well-characterized binding mechanisms that do not interfere with FcRn-mediated albumin recycling [8]. Linker design critically influences fusion protein success, with effects extending far beyond simple spatial connection. The (GGGGS)_3_ motif of 15 residues appears repeatedly in successful designs across diverse therapeutic proteins, providing a favorable balance between domain independence and manageable molecular weight for many applications [17]. However, linker optimization must be performed in the specific context of each therapeutic protein, as optimal length and composition depend on domain sizes, surface properties, and functional requirements.

Computational structure prediction using AlphaFold provides valuable but imperfect guidance for fusion protein design. Core helical regions of ABDs are predicted with high accuracy (backbone RMSD typically below 1 Å), sufficient for interface mapping and mutation planning. However, flexible regions such as long loop regions, some linker segments, and domain-domain orientations in multi-domain proteins show greater uncertainty that must be acknowledged in design strategies [92,93]. Confidence metrics (pLDDT and PAE scores) generally provide reliable indicators of prediction quality, enabling informed decisions about when predictions are trustworthy and when experimental validation is essential.

Remaining challenges include immunogenicity assessment requiring careful evaluation for each new therapeutic application, manufacturing complexity for fusion proteins incorporating therapeutic domains with complex disulfide patterns or post-translational modifications, and the optimization challenge inherent in multi-parameter design space where albumin-binding affinity must be balanced against therapeutic protein activity. Future directions include improved computational tools addressing current limitations in flexibility and ensemble prediction, novel de novo designed ABD variants optimized for specific applications without constraints of natural protein evolution, expansion to diverse therapeutic modalities including oligonucleotide therapeutics and peptide-drug conjugates, and personalized medicine applications leveraging the ability to tune half-life by modulating albumin-binding affinity.

ABD-fusion technology represents one valuable tool within a broader toolkit of half-life extension strategies including PEGylation, Fc fusion, and XTENylation. The optimal strategy depends on the size and properties of the therapeutic protein, the desired pharmacokinetic profile, manufacturing considerations, and intellectual property landscape. The integration of computational screening to rapidly eliminate problematic designs with experimental validation to confirm predictions and reveal unpredicted properties provides an efficient model applicable to diverse protein engineering challenges. By combining structural insight, computational prediction, and systematic experimental validation, the field is well-positioned to deliver the next generation of long acting biotherapeutics with enhanced efficacy and improved patient outcomes.

## Figures and Tables

**Figure 2.**
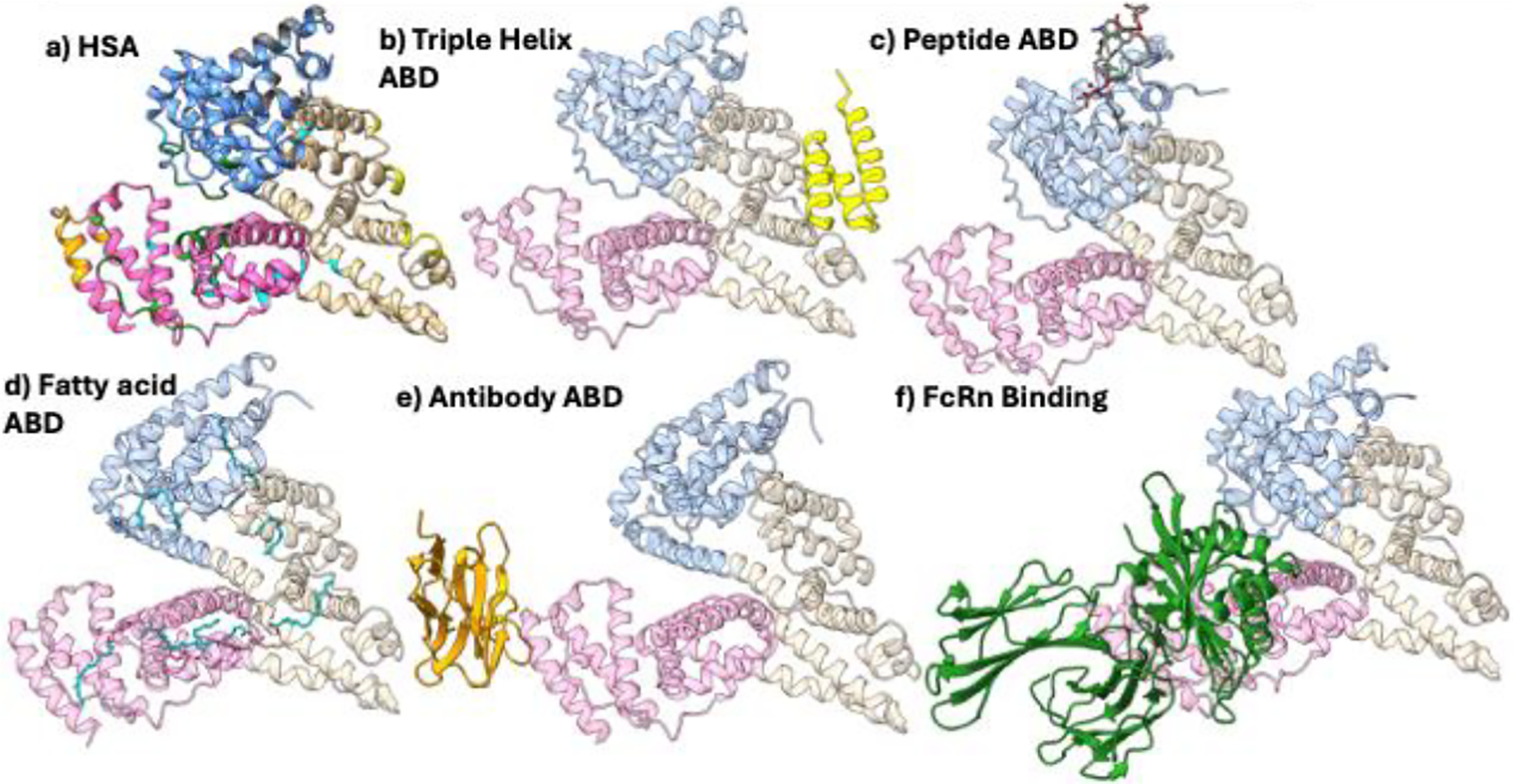
HSA in complex with various common ABDs. Each HSA protein is colored by domain: Domain I in blue, Domain II in tan, and Domain III in pink. a) HSA with the residues that bind to the selected ABDs colored as follows: bacterial three-helix in yellow, peptide based in gray, fatty acid in cyan, antibody derived in orange, and FcRn in green. b) The three-helix ABD derived from streptococcal protein G (PDB: 1TF0). c) The cyclic peptide dalbavancin in complex with HSA (PDB: 6M5E). d) Seven fatty acids bound to HSA (PDB: 1GNI). e) Nb.b201, a nanobody, bound to HSA (PDB: 5VNW). f) FcRn bound to HSA (PDB: 4N0F) [28–34].

**Figure 3.**
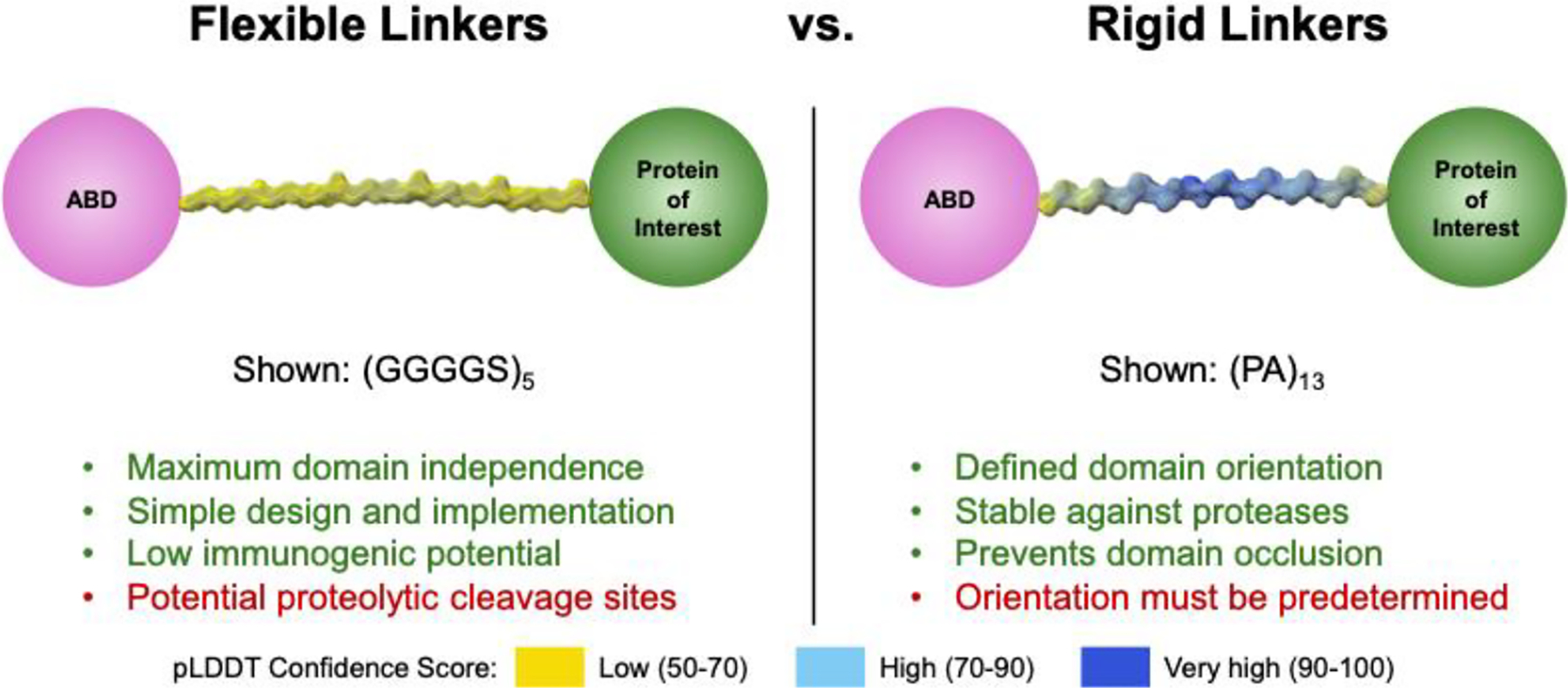
Comparison of flexible and rigid linkers. Linker structures were modeled on AlphaFold3 [54].

**Figure 4.**
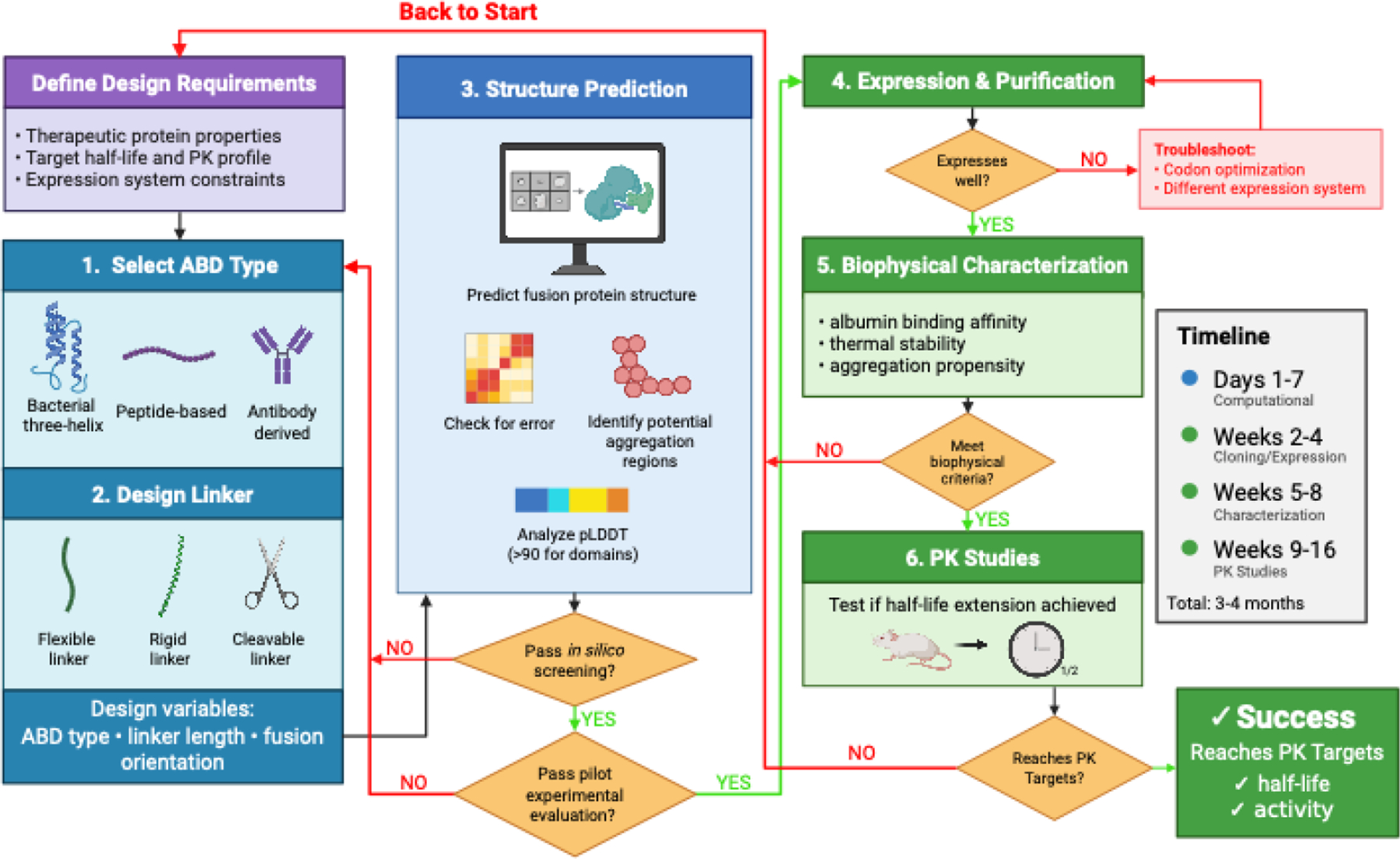
Iterative computational–experimental workflow for ABD fusion design. Rational design integrates ABD and linker selection, structure prediction with confidence metrics, biophysical characterization, and pharmacokinetic testing in an iterative optimization cycle. Created in BioRender. Bundy, B. (2026) https://BioRender.com/mw5o89w

**Table 1.** Comparison of albumin-binding domain classes used for half-life extension. Common albumin-binding domain classes are compared with respect to size, binding affinity, stability, cross-species reactivity, immunogenicity risk, and production considerations relevant to therapeutic protein fusion.

PROPERTY	BACTERIAL THREE-HELIX BUNDLE	PEPTIDE-BASED BINDERS	ANTIBODY-DERIVED
Size	5–7 kDa	<2 kDa, minimal peptides; 3–5 kDa lipid-conjugated peptides	12–15 kDa (VHH domains); variable for multivalent formats
Binding Affinity	Femtomolar to nanomolar	Micromolar to nanomolar	Picomolar to nanomolar
Thermostability	Excellent (Tm ~70–85°C)	Low to moderate	Good to excellent (~60–80°C)
Cross-Species Reactivity	Engineerable	Often-species dependent	Often species-specific; engineerable
Immunogenicity Risk	Low (after deimmunization)	Very low	Very low (humanized)
Production Cost	Low	Moderate to high (chemical synthesis required)	Moderate to high
Examples	ABDCon, ABD035, ABD094 [50]	Macrocyclic albumin-binding peptides; fatty-acid conjugates (liraglutide, semaglutide) [14,51]	Albumin-binding VHHs, knob domains, ozoralizumab [15,52]

**Table 2.** Comparison of linker architectures for albumin-binding fusion proteins. Flexible, rigid, and cleavable linker types used in ABD fusion proteins are compared, highlighting trade-offs in domain independence, conformational flexibility, stability, and functional control.

PROPERTY	FLEXIBLE (GGGGS)_N_	RIGID	CLEAVABLE
Domain Independence	Excellent	Limited	Excellent
Size Addition	Minimal (1–3kDa)	Minimal (1–2kDa)	Minimal (1–3kDa)
Design Complexity	Simple	Moderate	Complex
Proteolytic Stability	Variable	Good	Controlled
Best Application	General purpose fusions	Orientation-dependent binding	Conditional activation

## Abbreviations

The following abbreviations are used in this manuscript:

ABD: albumin-binding domains
GLP-1: glucagon-like peptide-1
HSA: human serum albumin
